# Bayesian Modeling Immune Reconstitution Apply to CD34+ Selected Stem Cell Transplantation for Severe Combined Immunodeficiency

**DOI:** 10.3389/fped.2021.804912

**Published:** 2022-02-15

**Authors:** Jean-Sebastien Diana, Naïm Bouazza, Chloe Couzin, Martin Castelle, Alessandra Magnani, Elisa Magrin, Jeremie Rosain, Jean-Marc Treluyer, Capucine Picard, Despina Moshous, Stéphane Blanche, Bénédicte Neven, Marina Cavazzana

**Affiliations:** ^1^Biotherapy Department, Hôpital Necker Enfants Malades, Assistance Publique-Hôpitaux de Paris, Paris, France; ^2^Université de Paris, Paris, France; ^3^Clinical Research Unit, Hôpital Necker Enfants Malades, Assistance Publique-Hôpitaux de Paris, Paris, France; ^4^Pediatric Hematology-Immunology-Rheumatology Unit, Hôpital Necker Enfants Malades Assistance Publique-Hôpitaux de Paris, Paris, France; ^5^Laboratory of Human Genetics of Infectious Diseases, Hôpital Necker Enfants Malades Assistance Publique-Hôpitaux de Paris, Paris, France

**Keywords:** Bayesian prediction algorithm, immune reconstitution, severe combined immunodeficiency (SCID), hematopoietic stem cell transplantation, CD34+ selection

## Abstract

Severe combined immunodeficiencies (SCIDs) correspond to the most severe form of primary immunodeficiency. Allogeneic hematopoietic stem cell transplantation (HSCT) and gene therapy are curative treatments, depending on the donor's availability and molecular diagnostics. A partially human leukocyte antigen (HLA)-compatible donor used has been developed for this specific HSCT indication in the absence of a matched donor. However, the CD34+ selected process induces prolonged post-transplant T-cell immunodeficiency. The aim here was to investigate a modeling approach to predict the time course and the extent of CD4+ T-cell immune reconstitution after CD34+ selected transplantation. We performed a Bayesian approach based on the age-related changes in thymic output and the cell proliferation/loss model. For that purpose, we defined specific individual covariates from the data collected from 10 years of clinical practice and then evaluated the model's predicted performances and accuracy. We have shown that this Bayesian modeling approach predicted the time course and extent of CD4+ T-cell immune reconstitution after SCID transplantation.

## Introduction

Severe combined immunodeficiencies (SCIDs) constitute a heterogeneous group of inherited disorders with a profound T-cell count reduction (1). Graft recipients with a matched sibling donor are curative and have the best clinical outcomes (2–4). However, in the absence of matched sibling donors, the optimal alternative donors and cell therapy strategies are subject to debate. Prolonged T-cell immunodeficiency observed after conventional T-cell graft depletion by CD34+ cell selection is a troublesome barrier to better clinical outcomes (5). Despite their immunodeficiency, SCID patients may manifest graft rejection or loss, and a small part of typical SCID will attain poor immune reconstitution (6). Recently published cohort studies suggest that a CD4^+^ T-cell count >500/mm^3^ in patients with SCID at 6 and 12 months after hematopoietic stem cell transplantation (HSCT) predicts long-term survival sustained immune reconstitution (7, 8). A challenging problem in this domain is giving value to interpreting the early time point of T-cell reconstitution. Therefore, a new approach is needed to make a faster and more robust analysis of post-transplant immune reconstitution. The aim here is to investigate a modeling approach to predict the time course and extent of CD4+ T-cell immune reconstitution after SCID transplantation. A mixed-effects framework and early post-HSCT data can usefully obtain the approximate Bayesian computation for individual children's long-term reconstitution trajectories. We will test the algorithm's performance to compute faster immune reconstitution analysis.

## Materials and Methods

### Data

In a retrospective study between January 2008 and December 2017, we included 32 consecutive patients with SCID having received a primary HLA-haploidentical CD34+ selected graft from a family member. Patients with a diagnosis of adenosine deaminase deficiency and intrathymic deficiency were excluded. The dataset used for model building and covariate analysis was collected during routine clinical practice in the Immunohematology and Rheumatology Department at Necker Children's Hospital (Paris, France). The myeloablative conditioning regimen (CR, when used) was based on a combination of busulfan, fludarabine (160 mg/m^2^), and serotherapy [5 or 10 mg/kg anti-thymocyte globulin (ATG) or 1 mg/kg alemtuzumab] (9). Only four patients treated before 2010 underwent myeloablative CR with a CY-BU myeloablative conditioning protocol (10). In the Omenn syndrome, we initiated a 3-month therapeutic course with cyclosporine for most cases. All patients and donors gave their written informed consent to the collection and anonymous analysis of HSCT-related data.

### Predicting Reconstitution From Early Data and Individual Covariates

The mechanistic inference model of the CD4+ T-cell count was based on naive CD4+ T-cell homeostasis, age-related changes in the thymic output, and the cell proliferation/loss model optimized from the Hoare et al. model (11, 12).

The complete model from Hoare et al. can be summarized as follows:


$$\begin{array}{l} \frac{dX}{dt}=\lambda -dX+pX \end{array}$$

Λ represents the thymic output of T cells and was coded as λ(*t*, τ) = λ_0_λ_*age*_Δ_*HSCT*_(*t*); λ_0_ corresponds to the theoretical thymic output (cell/days); λ_age_ stands for the age-scaling function of the thymic output; Δ_HSCT_(*t*) stands for the sigmoidal function to recover the thymic output (including both the time to recovery of the thymic output and the rate of recovery in thymic output parameters). The parameter *p* represents the proliferation rate and was coded as $p\left(X,\;t,\;\tau \right)=y\left(\tau \right)p_{0}e^{\left(1-\frac{X\left(t\right)}{N\left(t\right)}\right)}$, where *p*_0_ stands for the theoretical proliferation rate (/days); *X(t)* stands for CD4 T-cell concentration with time *t* after HSCT; *N*(τ) stands for the expected total CD4 for a healthy child of age τ days; and *y*(τ) is the proportion of CD4 cells expressing Ki67 as a function of age. The parameter *d* stands for the cell loss rate and was coded as $d\left(X,\;t,\;\tau \right)=y\left(\tau \right)d_{0}e^{\left(\frac{X\left(t\right)}{N\left(t\right)}-1\right)}$, where *d*_0_ stands for the theoretical cell loss rate (/days). This mechanistic non-linear mixed-effect model was applied to our data, and model parameters were estimated using Monolix software (version 2019, Lixoft, Antony, France) (13). The Monolix code is provided in the Supplementary Data.

We chose eight specific pre-HSCT covariate parameters that putatively influence CD4+ T-cell reconstitution after CD34+ selected stem cell transplantation: age at transplantation, the type of molecular defect, Omen syndrome, pre-HSCT viral diseases, the ATG dose level (5 or 10 mg/kg), the presence or absence of a CR, and the CD34+ or CD3+ count of the stem cell graft. Model parameters were estimated using the stochastic approximation expectation–maximization and Markov chain Monte Carlo algorithm. The Fisher information matrix and the likelihood were computed using stochastic approximation and importance sampling. We also used the likelihood ratio test (including the log-likelihood), Akaike information criterion, and Bayesian information criterion to test different hypotheses regarding the covariate effects on parameters, the residual error model (additive, proportional, or combined), and the structure of the variance–covariance matrix between-subject variability parameters. A sensitivity analysis was also conducted by excluding patients with “other” diagnosis, and model parameters were re-estimated.

The model's goodness of fit was evaluated by a visual inspection of the observed–predicted confrontation (population and individual) scatter plots. Diagnostic graphics and other statistics (including the visual predictive check and normalized prediction distribution errors) were generated using R software (14).

### Final Bayesian Model

We simulated different reconstitution profiles with one data point per month (1,000 Monte Carlo simulations from the final model) (15). The simulated CD4+ T-cell count at 12 months was predicted (using Bayesian forecasting) from 3- or 6-month truncated simulated trajectories. The probability of a correctly predicted CD4+ T-cell count <500/mm^3^ at 12 months defined the sensitivity. The method's specificity was defined as the probability of correctly predicting a CD4+ T-cell count >500/mm^3^ at 12 months. Our model's accuracy was determined by the overall probability of correctly classifying a patient with a CD4+ T cell > or <500/mm^3^ at 12 months.

## Results

### Characteristics of Patients

**Figure 1:**
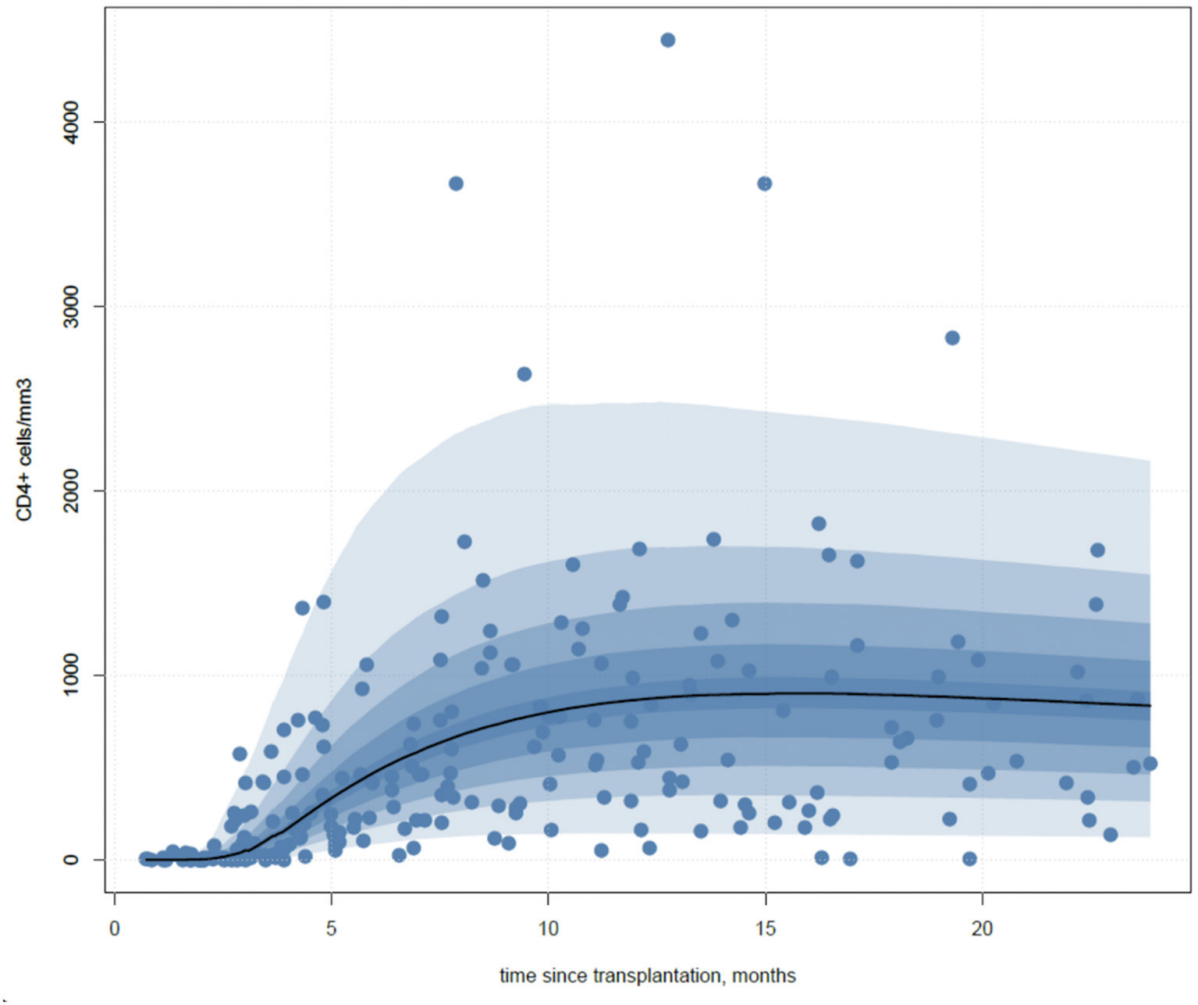
Immune reconstitution model. The prediction distribution from the final model. The blue area corresponds to the 95% prediction interval (PI). Blue points correspond to data for CD4+ T-cell reconstitution after HLA-haploidentical, CD34+ selected HSCT.

A total of 231 data points for the T-cell phenotype of 32 consecutive patients were available for the 2 years following HSCT and were included in the immune reconstitution model (Figure 1). We obtained a median of seven samples per patient (range: 3–23 samples) over a median follow-up period of 20 months (range: 3–24). The patients' characteristics and outcomes are summarized in Table 1. Five of the patients (15.6%) died during the study period: four from infectious diseases and one from GvHD, followed by an infection. Two patients did not engraft, and the cumulative incidence of acute grade ≥II GvHD was 20.5%.

**Table 1:** Patient characteristics.

	**IL2RG/ JAK3 deficiency**		**RAG1, ARTEMIS deficiency**		Other deficiencies*,^**^⋆⋆⋆^**^	
	**(*****n*** **=** **17)**		**(*****n*** **=** **11)**		**(*****n*** **=** **4)**	
Age (years)	0.52	(0.004–1.61)	0.38	(0.25–1.13)	0.62	(0.20–0.95)
Immediate pre-HSCT morbidities*						
Omenn syndrome	1	(5.88%)	4	(36.36%)	1	
Infectious diseases	3	(17.65%)	3	(27.27%)	2	
Conditioning regimen	6	(35.29%)	10	(90.9%)	3	
**Stem cell transplant**						
106 CD34+/kg	12.75	(1.89–26.6)	9.63	(4.00– 17.85)	13.67	
CD3+/kg	2,630	(690–5,340)	3,587	(196–3,510)	3.850	
**Outcomes**						
Death	1	(5.88%)	3	(27.7%)	1	
Viral disease	2	(11.76%)	3	(27.7%)	0	
BCGitis^‡^	1	(5.88%)	1	(9.09%)	0	
Acute GvHD	4	(23.53%)	3	(27.7%)	1	
Autoimmune disease	0	(0.00%)	1	(9.09%)	1	
**CD4+** **T-cell count (/mm**^**3**^**)**						
3 months	19	(0–256)	22	(0–576)		
6 months	448	(288–1,056)	302	(99–462)		
12 months	747	(52–1,386)	444	(60–4,444)		

*Clinical status before hematopoietic stem cell transplantation.

*^‡^Systemic bacillus Calmette–Guérin infection confirmed by bacterial culture.*

*^⋆^^⋆^^⋆^Other deficiencies: CD3e deficiency, ZAP70 deficiency, and reticular dysgenesis*.

### Model Fitting

The one-compartment turnover model appeared to be suitable for comparing and assessing the time course of CD4+ T-cell reconstitution after HSCT. The thymic output increased from 2.7 months after HSCT [95%CI: (2.3–3.2)]. After transplantation, CD4+ T-cell counts had a lower distribution than age-appropriate reference. Interestingly, the 95th percentile of the observed values appeared within the normal range.

**Figure 2:**
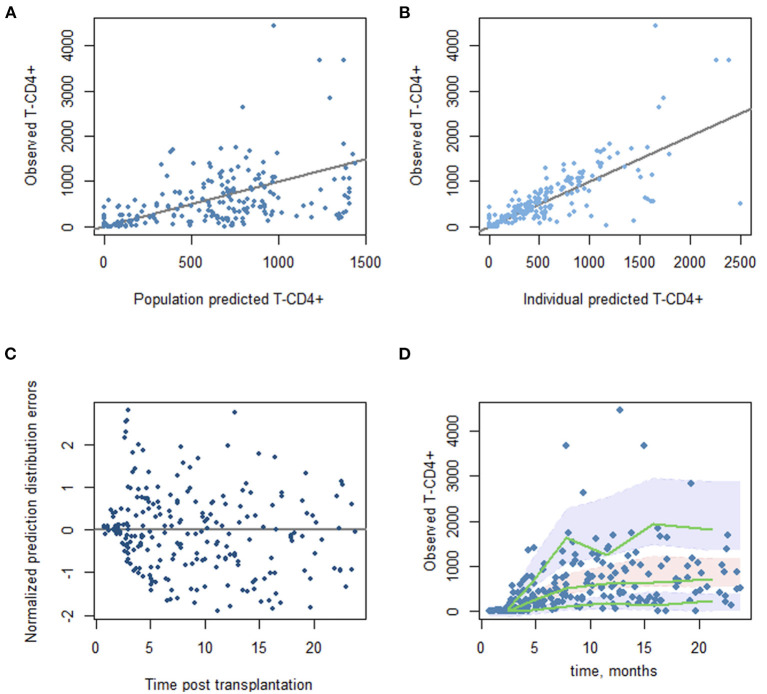
Diagnostic plots from the final mode. **(A)** Scatter plot of the observed vs. predicted population CD4 T-cell counts. **(B)** Observed vs. predicted individual CD4 T-cell counts. **(C)** Normalized prediction distribution errors vs. time since transplantation. **(D)** Prediction-corrected visual predictive check plots. The green lines correspond to the observed data's 5th, 50th, and 95th percentiles. The shaded areas represent the 95% confidence interval around the simulated percentiles.

Observed vs. predicted CD4+ T-cell counts were close with the median (range) values, respectively, of 35 (0–576) vs. 35 (0–389) at 3 months, 414 (99–1,056) vs. 566 (108–1,059) at 6 months, and 564 (52–4,444) vs. 577 (67–2,661) at 12 months. The model parameters and diagnostic plots of the model's performance are provided in Figure 2.

### Pre-HSCT Covariates Parameters

Genetic diagnosis and CR were the two pre-transplant covariates significantly associated with the time-course of T-cell reconstitution (*p* = 0.0047 and 0.01, respectively). Relative to patients with an IL2RG/JAK3 defect, patients with a RAG1 or ARTEMIS deficiency or another diagnosis had a 36.8% [95%CI: (18.3–73.6)] decrement in their thymic output function. Otherwise, CR significantly decreased the cell loss rate function [43% (22.5–82.1)]. The parameter estimates for the T-cell concentration at the time of HSCT were not influenced by ATG dosing, immediate pre-HSCT morbidities, or the stem cell graft's composition (Table 2). The sensitivity analysis excluding patients with “other” diagnosis showed that all structural parameters were very close to the primary analysis, suggesting the model's robustness. Furthermore, this sensitivity analysis showed that genetic diagnosis and CR were still significantly associated with the time course of T-cell reconstitution (*p* = 0.0396 and 0.015, respectively), supporting the primary analysis. The model parameters estimated from this sensitivity analysis are provided in the Supplementary Data.

**Table 2:** Population parameter estimates.

**Fixed effects**	**Estimate**	**SE**.	**RSE (%)**
Time to recovery thymic output (days)	82.3	7.51	9.12
Rate of recovery in thymic output*	10	Fixed	
Theoretical thymic output (cell/days)	0.279	0.0809	29.1
Effect size of no IL2RG/JAK3 defect on thymic output^§^	−1	0.354	35.4
Theoretical cell loss rate (/days)	2.09	0.562	26.9
Effect size of conditioning regimen on theoretical cell loss^§^	−0.844	0.33	39.1
Theoretical proliferation rate (/days)*	0.207	Fixed	
**Standard deviation of the random effects**			
Omega_theorical thymic output	0.708	0.191	27
Omega_time to recovery thymic output	0.209	0.0825	39.5
Omega_theorical cell loss rate	0.619	0.17	27.5
**Error model parameters**			
a	82.6	8.21	9.93
b	0.482	0.0387	8.03

SE, standard error; RSE%, relative standard error (SE/estimate ^*^ 100). Standard deviation of the random effect: omega, between-subject variability estimates on thymic output, time to recovery of the thymic production, and cell loss rate. Error model parameters: a and b, residual additive and proportional variabilities estimates, respectively.

*^§^Significant categorical covariates are included through the multiplication of the parameter by exp (effect size).*

*Rate of recovery in thymic output and theoretical proliferation rate were fixed to Hoare et al. values.

**Figure 3:**
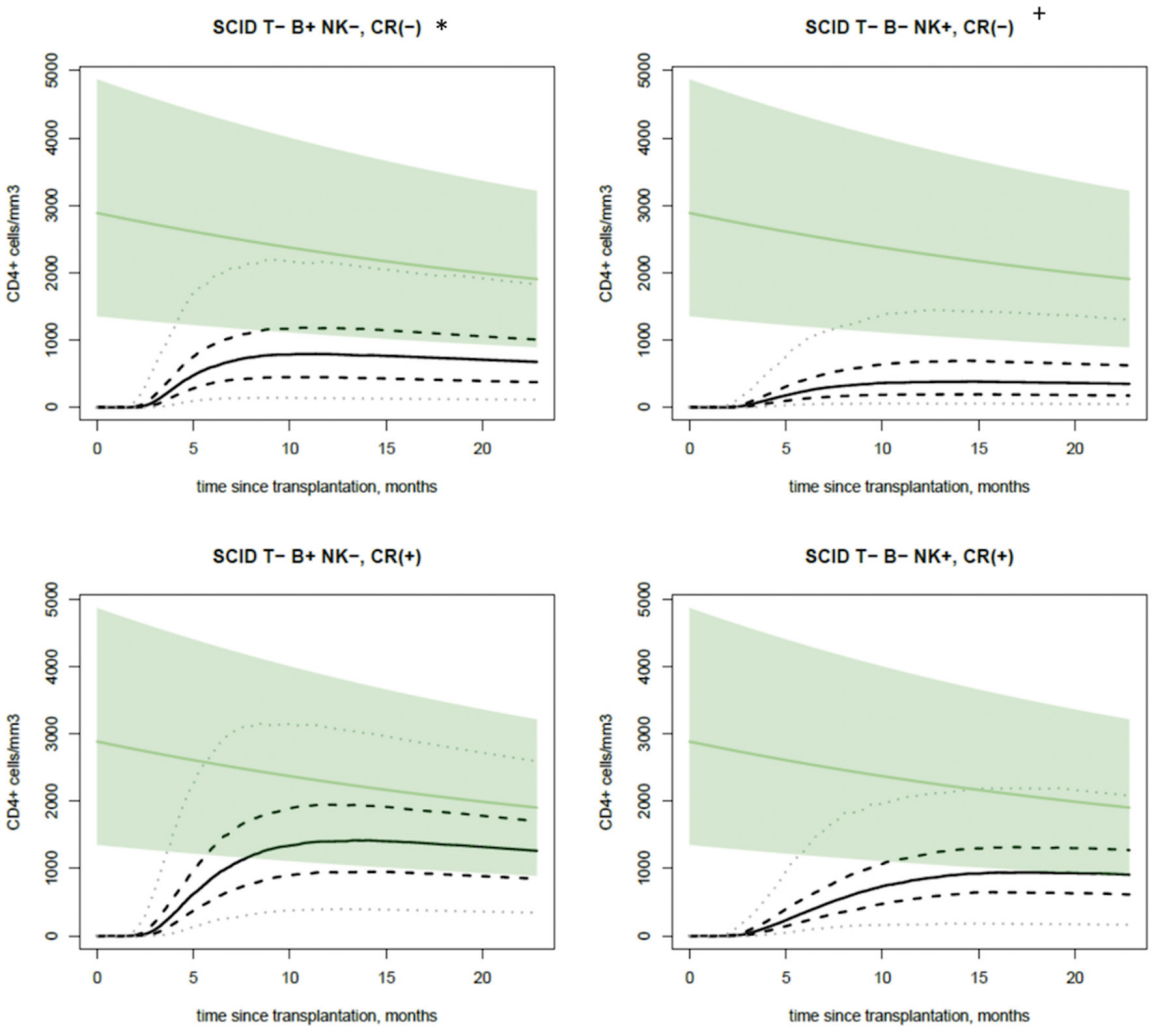
Immune reconstitution profiles. The PI for the simulated change over time in the CD4+ T-cell count time course for a child aged 6 months at the time of HSCT, as a function of significant covariate effects in the model (i.e., the presence or absence of a molecular defect and the presence or absence of conditioning). The continuous, dashed, and dotted black lines correspond to the median, 50%PI, and 95%PI, respectively. The green line and green area correspond to the age-appropriate reference value and the 95% lower and upper bound.

Regarding covariate, our model provided four theoretical T-CD4+ immune reconstitution curves for SCID following HSCT with CD34+ selection: SCID T-B+ NK- or SCID T-B-NK+ with or without a CR (Figure 3). In addition, simulated change over time in the CD4+ T-cell count crossed into the normal reference range depending on the subgroup, especially for T-B+ SCID patients who received conditioning.

### Predicting Reconstitution From Early Data and Individual Covariates

The population parameter means and variances found from the initial model fitting were used as the final Bayesian model's priors. For 1,000 simulated individuals, the overall accuracy in predicting the CD4+ reconstitution at 12 months with trajectories truncated at 3 and 6 months was 79 and 87%, respectively. The course truncated at 3 months had a low estimated sensitivity (41%) but very high specificity (88%). In contrast, the simulated trajectory truncated at 6 months was substantially more sensitive (74%) and still highly specific (90%).

## Discussion

Bayesian methods are becoming increasingly important in the biological sciences for inferring cellular networks, modeling biology systems, and integrating medical data (16, 17). Bayesian computation may provide significant benefits in terms of early post-transplant immune reconstitution investigation. For example, after stem cell transplantation for SCID, competent and fast immune reconstitution is the primary prognostic criterion, especially when considering severe pre-transplant infectious diseases.

The model fitted well with our retrospective data. Unfortunately, cross-validation was hard to provide in the case of rare diseases. Nevertheless, our modeling results were interestingly in line with the data from previous cohort studies (18). Computation reflected, in part, the physiopathology of post-HSCT CD4+ T-cell reconstitution for SCID. RAG1 and Artemis deficiencies are characterized by a defect of V(D)J recombination activity and altered thymic stroma, and T-B-NK+ SCID was associated in the model with a lower thymic output (19). On the other hand, CR decreased the cell loss rate function, suggesting better engraftment of a T-cell precursor.

As a prediction model, we decide to use only pretransplant covariates. Therefore, moderate-to-severe GvHD or post-transplant immune-suppressive treatment was not considered. Otherwise, the myeloid engraftment is associated with improved immune reconstitution, including long-term T-cell reconstitution. In recent years, there has been significant interest in exploring busulfan exposures in the sub-myeloablative range as a compromise between efficacy and safety and a guide for making treatment decisions. Unfortunately, we could not recover the data for BU exposure for all patients to implement our model. In addition, ATG pharmacokinetic studies were not performed in routine during this time of the study.

Other modeling strategies, based on multivariate immune reconstitution patterns, identified variables and described the correlation between immune system dysfunction and the occurrence of complications. Principal component analysis is a performant tool and is valuable for the simultaneous interpretation of different parameters (20, 21). However, those models need an exhaustive post-transplant multiparameter database, which is not adapted to rare diseases. Furthermore, they do not use single factors, and neither elaborate prediction adjusts to clinical practice.

A low number of patients, large dataset, and multiparameter studies do not also fit with conventional statistical methods, in which problems with mass significance can quickly occur. In contrast to a comparative statistical test at a single time point, the model provides a dynamic assessment of immune reconstitution while increasing substantially (by up to 10-fold) the ability to detect covariate effects. Our model also counts the variability in the number of data points per patient. In addition, the representation and use of the probability theory make Bayesian computation suitable for combining domain knowledge and data, avoiding to overfit a model to training data, and learning from incomplete datasets. The establishment of the model and parameter allowed us to predict a probability of distribution of T-CD4+ for each subpopulation. For example, in the T-B- group, nearly everyone received conditioning. However, only 1/11 patients were unconditioned due to pre-transplant severe comorbidities and a high risk of toxicity. Therefore, the Bayesian method did not consider only one patient but interpreted all data points with model and covariate adjustments to compute the curve of IR for the T-B- group.

However, a larger dataset may have also allowed us to point out other covariates. It could explain the remaining between individual variability and substantially. A larger data set may also increase the model's ability to predict personal CD4+ reconstitution very soon after transplantation. Post-transplant clinical events had a low incidence in our cohort. Contrary to other studies, we were not able to report the different immune reconstitution of T-CD4+ before the onset of GvHD, post-transplant viral infectious disease, or BCGitis (22).

The Bayesian inference is a data-driven technique; the prior probabilities of an event are updated when the new data are gathered. It was able to identify a relatively high proportion of satisfactorily simulated trajectories very soon after transplantation (i.e., within 3 months). A repetitive time point of phenotyping T cell until 6 months should be required to increase sensitivity and specificity substantially. CD4+ T-cell immune reconstitution is widely used and is a strong predictor of event-free survival (EFS), overall survival (OS), viral diseases, and reactivation or non-relapse mortality (NRM), depending on stem cell transplant indications (23–25). Only a few models attempt to predict T-CD4+ cell reconstitution (12, 26). In the context of new cell therapy approaches for T-cell depletion or *in vitro* thymic maturation, the mechanistic model presented here could be used to design clinical trials and provide early assessments of their results. Furthermore, it should be an exciting model to compare immune reconstitution from a different group of patients statistically. The Bayesian modeling approach predicted the time course and extent of CD4+ T-cell immune reconstitution after SCID transplantation.

## Data Availability Statement

The raw data supporting the conclusions of this article will be made available by the authors, without undue reservation.

## Author Contributions

J-SD and NB conceptualized, designed the study, and drafted the initial manuscript. MC performed the supervision of this work. All authors reviewed, revised, and approved the final manuscript as submitted and agreed to be accountable for all aspects of the work.

## Conflict of Interest

The authors declare that the research was conducted in the absence of any commercial or financial relationships that could be construed as a potential conflict of interest. The Reviewer SP has declared a past collaboration with several of the authors DM and BN to the handling editor.

## Publisher's Note

All claims expressed in this article are solely those of the authors and do not necessarily represent those of their affiliated organizations, or those of the publisher, the editors and the reviewers. Any product that may be evaluated in this article, or claim that may be made by its manufacturer, is not guaranteed or endorsed by the publisher.
