# Supplementary material for: Bayesian Modeling Immune Reconstitution Apply to CD34+ Selected Stem Cell Transplantation for Severe Combined Immunodeficiency
Source: Front Pediatr. 2022 Feb 15;9:804912. doi: 10.3389/fped.2021.804912 (PMC8885722; doi:10.3389/fped.2021.804912)
Supplement: Supplementary file 1 [file Data_Sheet_1.docx]

**Supplemental data file 1. Monolix software code.**

[LONGITUDINAL]

input = {TVL, TVD, TVPR, TVINT, TVLH, TVLR, XAGE}

XAGE = {use=regressor}

EQUATION:

LAMBDA0=TVL

DELTA0=TVD

PROL0=TVPR

INT=TVINT

LH=TVLH

LR=TVLR

CD4_0 = INT

VAGE = t + XAGE

VT = 924 + 2354*exp(-0.001012*VAGE)

V = 496.5 + 2074*exp(-0.000869*VAGE)

LLL = LAMBDA0* 0.02*exp(-0.00027*VAGE) * V / 0.0221 * 0.02

DD = DELTA0* 0.02*exp(-0.00027*VAGE)

PRR = PROL0* 0.02*exp(-0.00027*VAGE)

ddt_CD4 = LLL * (1-exp(-2*t/LH))/(1+exp(LR*(1-t/LH))) - CD4*(DD*exp(CD4/VT -1) - PRR * exp(-CD4/VT+1))

OUTPUT: output = {CD4}

**Supplemental data file 2. Population parameters estimates (excluding patients with ‘other’ diagnosis).**

| **Fixed Effects** | | **Estimate** | **S.E.** | | **R.S.E. (%)** |
| --- | --- | --- | --- | --- | --- |
| Time to recovery thymic output (days) | | 83.9 | 9.71 | | 11.6 |
| Rate of recovery in thymic output* | | 10 | fixed | |  |
| Theoretical Thymic output (cell/days) | | 0.284 | 0.113 | | 39.9 |
| *Effect size of no IL2RG/JAK3 defect on thymic output^§^* | | -0.863 | 0.42 | | 48.6 |
| Theoretical cell loss rate (/days) | | 2.17 | 0.722 | | 33.3 |
| *Effect size of conditioning regimen on theoretical cell loss^§^* | | -0.885 | 0.364 | | 41.1 |
| Theoretical proliferation rate (/days)* | | 0.207 | fixed | |  |
| **Standard Deviation of the Random Effects** | | |  | |  |
| omega_Theorical Thymic output | 0.739 | | 0.245 | | 33.2 |
| omega_Time to recovery thymic output | 0.251 | | 0.121 | | 48.3 |
| omega_Theorical cell loss rate | 0.621 | | 0.213 | | 34.3 |
| **Error Model Parameters** | | | | | |
| **a** | | 79.8 | 8.36 | 10.5 | |
| **b** | | 0.501 | 0.0431 | 8.59 | |

Key : S.E, Standard error; R.S.E%, relative standard error (S.E / estimate*100). Standard Deviation of the random effect: omega, between-subject variability estimates on Thymic output, time to recovery of the thymic production, and Cell Loss Rate. Error model parameters: a and b, residual additive and proportional variabilities estimates, respectively. ^§^Significant categorical covariates are included through the multiplication of the parameter by exp (Effect size).
